# Genomic selection accuracies within and between environments and small breeding groups in white spruce

**DOI:** 10.1186/1471-2164-15-1048

**Published:** 2014-12-02

**Authors:** Jean Beaulieu, Trevor K Doerksen, John MacKay, André Rainville, Jean Bousquet

**Affiliations:** Natural Resources Canada, Canadian Forest Service, Canadian Wood Fibre Centre, 1055 du P.E.P.S, Stn. Sainte-Foy, P.O. Box 10380, Quebec City, QC G1V 4C7 Canada; Canada Research Chair in Forest and Environmental Genomics, Institute for Systems and Integrative Biology, Université Laval, Quebec City, QC G1V 0A6 Canada; Ministère des Forêts, de la Faune et des Parcs du Québec, Direction de la recherche forestière, 2700 rue Einstein, Quebec City, QC G1P 3 W8 Canada

**Keywords:** Genomic selection, Genotype-by-environment interactions, Relatedness, Somatic embryogenesis, Spruce

## Abstract

**Background:**

Genomic selection (GS) may improve selection response over conventional pedigree-based selection if markers capture more detailed information than pedigrees in recently domesticated tree species and/or make it more cost effective. Genomic prediction accuracies using 1748 trees and 6932 SNPs representative of as many distinct gene loci were determined for growth and wood traits in white spruce, within and between environments and breeding groups (BG), each with an effective size of *N*_*e*_ ≈ 20. Marker subsets were also tested.

**Results:**

Model fits and/or cross-validation (CV) prediction accuracies for ridge regression (RR) and the least absolute shrinkage and selection operator models approached those of pedigree-based models. With strong relatedness between CV sets, prediction accuracies for RR within environment and BG were high for wood (*r* = 0.71–0.79) and moderately high for growth (*r* = 0.52–0.69) traits, in line with trends in heritabilities. For both classes of traits, these accuracies achieved between 83% and 92% of those obtained with phenotypes and pedigree information. Prediction into untested environments remained moderately high for wood (*r* ≥ 0.61) but dropped significantly for growth (*r* ≥ 0.24) traits, emphasizing the need to phenotype in all test environments and model genotype-by-environment interactions for growth traits. Removing relatedness between CV sets sharply decreased prediction accuracies for all traits and subpopulations, falling near zero between BGs with no known shared ancestry. For marker subsets, similar patterns were observed but with lower prediction accuracies.

**Conclusions:**

Given the need for high relatedness between CV sets to obtain good prediction accuracies, we recommend to build GS models for prediction within the same breeding population only. Breeding groups could be merged to build genomic prediction models as long as the total effective population size does not exceed 50 individuals in order to obtain high prediction accuracy such as that obtained in the present study. A number of markers limited to a few hundred would not negatively impact prediction accuracies, but these could decrease more rapidly over generations. The most promising short-term approach for genomic selection would likely be the selection of superior individuals within large full-sib families vegetatively propagated to implement multiclonal forestry.

## Background

Genomic selection GS, [1] has been proposed as an alternative to conventional pedigree-based selection (PS), where pedigree information is replaced with dense genetic marker information to estimate the genetic worth of each individual. In GS, thousands of markers are modeled simultaneously and the marker effects are summed, in order to compute the genomic (GEBV) equivalent of estimated additive genetic (breeding) values (EBV). GS allows small-effect loci to be captured, obviating significance testing in association and linkage studies. PS has been very effective at capturing the parent average component of the EBV, but cannot capture variation in the proportion of genome shared by pairs of relatives due to Mendelian sampling in the absence of an individual’s own phenotypic record or those of its descendants [2, 3]. Conversely, GS can theoretically capture the Mendelian sampling component in the absence of recorded phenotypes because at an appropriate marker density, all loci that explain some of the phenotypic variation are presumably tagged.

Theoretical [4–6] and empirical studies in breeding populations of forest trees [7–9] and other long-generation plant species [10, 11] have shown that substantial genetic gains can be achieved through GS. It came out that the main advantage of GS over conventional breeding in long-lived species was the possibility to reduce the generation time through the selection based on marker effects before phenotypes are available. Thus, any (possible) loss of EBV precision could be compensated for by completing additional (faster) cycles of recurrent selection. However, less attention has been paid to the effect of relatedness between cross-validation (CV) training and testing sets, choice of model and/or the possibility of predicting in new, unknown environments.

The main assumption of GS is that marker coverage is sufficiently dense so that linkage disequilibrium (LD) between markers and causal loci will not be considerably broken up following recombination/meiotic events. One way to increase the chance that markers are in LD with causal loci is to reduce the effective population size (*N*_*e*_), as it is easily controlled by the plant and tree breeders [4]. It has been shown that the increase in prediction accuracy with a reduction of *N*_*e*_ is striking at lower marker densities, but minor at higher marker densities [4]. Thus, one could expect that when working with a population of small effective size, a small number of markers might be sufficient to capture the LD and obtain high prediction accuracies. However, reducing *N*_*e*_ also increases within-population relatedness, and because markers, even at low density coverage, can capture close relatedness between individuals in the training (estimation) and testing (validation) sets, EBV predictions in CV testing sets can be non-zero despite the absence of LD [12]. Indeed, empirical studies removing relatedness between training and testing sets at the level of the population (breed) [13], subpopulation [14] or family [15–17] levels, including a previous study on white spruce [18], found predictive abilities to be drastically reduced or to fall to zero. Similarly, it was suggested that predictive accuracy was driven by markers tracing family relatedness in pine [19, 20]. This stresses the importance of designing training and testing sets appropriate to the conditions under which GS might be used [17] so that correct conclusions can be drawn and, also, so that marker densities that make it possible to capture not only relatedness but also LD can be used.

Model choice may be important insofar as it must align with the genetic architecture of the trait. Ridge regression (RR) [21, 22] is a model appropriate for traits controlled by a large number of small-effect loci, such as growth and wood traits e.g. [23–25], closely resembling assumptions of the infinitesimal pedigree-based (polygenic) model. Methods such as BayesA/BayesB [1] or least absolute shrinkage and selection operator (LASSO) [26] are more relevant for traits controlled by few(er) genes of large(r) effect and are supposedly better at zeroing out (shrink) loci of negligible effect [27]. The simpler, more parsimonious RR is widely used in GS as it often performs as well as more complex models e.g. [9, 28], even though RR is known to better capture relatedness [12], which complicates the interpretation of its effectiveness.

So far, GS prediction accuracy has mostly been evaluated within single environments. However, as reforestation programs take place on a variety of sites, genotype-by-environment (GE) effects can be substantial and should be considered. It has been shown that a multivariate model, where traits measured on different sites are treated as different correlated traits, could improve predictive ability (correlation between observed and predicted phenotype) for lines tested in one environment but not in the other, thus capitalizing on correlated information between environments [29]. However, this model was less powerful for a line not tested in any of the two environments, with predictions drawn solely from information on relatives [29]. Similarly, univariate prediction accuracies dropped when marker effects estimated in a given environment were used to make predictions in different environments [7, 14], although a recent study on white spruce in eastern Canada showed that prediction accuracy could only be slightly reduced in such situations [18]. Thus, given species-specific considerations and the potential variability of the reforestation landscape, it appears important to determine whether the estimated marker effects have accurate predictive value in the different reforestation environments.

The objectives of this study were 1) to evaluate how type of model, relatedness between training and testing sets, marker subsets, and across-environment predictions affect model fit or prediction accuracy for wood and growth traits, 2) to estimate the relative efficiency of marker-based versus pedigree-based models, and 3) to discuss implications for the use of GS in the context of spruce improvement and its long breeding cycles.

## Methods

### Data

#### Phenotypes

Genetic material was sampled from a larger test series (E952) designed to assess the genetic merit of first-generation selections, which had been subdivided into breeding groups delineated by their provenance or geographic region [30]. Crosses were made using a partial diallel mating design within each of six distinct breeding groups (BG) or sublines [31], which were designed to limit future inbreeding to within group. Each parent was used in crosses 1–3 times, giving rise to a mixture of full- and half-sib families within breeding groups. Genetic tests were established in 1997 from 2-year-old nursery-grown seedlings near Asselin Township (S1-C, located in the balsam fir–yellow birch ecological zone; cool weather), and St. Casimir (S2-D, located in the maple–basswood ecological zone; warm weather), Québec, Canada, in a randomized complete block design. Trees were assigned to row-plots of five trees/plot (2 m × 2 m spacing).

For the current study, the base population (*n* = 39) consisted of 19 parents in a first breeding group (BG1) and 20 parents in a second (BG2) with 27 and 32 full-sib crosses (59 total) made within each group, respectively. The sampled trees were drawn from 16 blocks over the two sites and 25–33 individuals were sampled per full-sib cross.

In 2012, wood cores were extracted from 17-year-old trees with a diameter at breast height (DBH17) ≥ 5 cm on both sites for determination of wood density (ADEN) and microfibril angle (AMFA). AMFA was measured by scanning the radial face of the last complete growth ring closest to the bark with a D8 Discover X-ray diffractometer (Bruker AXS, Madison, WI, USA). Diffraction profiles of a few samples were first calibrated by comparison with values obtained using compound light microscopy before the complete population of samples was scanned. Density was determined by scanning increment cores from pith to bark along the radial face at a resolution of 25 μm in the QTRS-01X Tree Ring Analyzer (Quintek Measurement Systems Inc., Knoxville, TN, USA). A weighted mean density trait was obtained by summing the product between annual density measures and the ratio of respective radial areas to total radial area. Before collecting wood cores, trees were measured for age 17 height (HT17) and diameter (DBH17) growth. Phenotypes outside ±3 phenotypic standard deviations following adjustment for fixed block effects were considered as outliers and set to missing. A total of 1,748 offspring, with at least one non-missing record among the four traits, were retained after cleaning the genotype data (see below); i.e. 851 and 897 from sites S1-C and S2-D, 841 and 907 from breeding groups BG1 and BG2, respectively (Table 1).

**Table 1:** **Number of sampled trees across test sites (S) and breeding groups (BG)**

Site*	BG1	BG2	Total
S1	411	440	851
S2	430	467	897
Total	841	907	1,748

*S1: Asselin Township (S1-C); S2: St. Casimir (S2-D).

#### Genotypes

Parents (n = 39) and progeny (n = 1,748) were genotyped with the white spruce Infinium SNP array PgLM3 [32] at a rate of about one SNP per target gene locus. SNP loci were cleaned by removing those loci that failed; they were monomorphic, displayed a call rate < 0.85, had a minor allele frequency (MAF) < 0.005 (less than 10 heterozygous individuals), or a mismatch with both parental genotypes. A total of 6,932 high quality SNPs with a mean MAF = 0.20 were retained for GS analyses. These SNPs were distributed among 6,918 distinct gene loci. Individual genotypes with call rates < 0.80 were removed. Missing genotypes (~ 2.7%) were imputed from the binomial distribution, with success probabilities given the observed allele frequencies.

In order to test for population structure and control for relatedness as an alternative to the numerator relationship matrix (NRM) for association testing, an identity by state (IBS), allele-sharing, realized genomic relationship matrix (GRM, ***K***) was constructed as:
1

where *M* is an *n* × *m* (individuals × loci) matrix of SNPs coded as 0, 1 and 2 for genotypes 11, 12 and 22, respectively, and *P* is an *n* × *m* matrix with columns containing the frequency of the second allele *p*_*m*_ (here the minor allele) at locus *m*. Subtracting *P* from *M* gives *Q*, which sets mean values of the allele effects (and thus BVs) to zero. Finally, dividing *QQ*^*T*^ by *k* = *tr (QQ*^*T*^*)/m* (i.e. the mean of the diagonal) normalizes the GRM to a scale analogous to elements in the *A* matrix [33]. To test for population structure, the *K* matrix (= *VDV*^T^) was decomposed spectrally with the eigen() function in R v2.15.2 [34], where *D* is a diagonal matrix of eigenvalues and columns of ***V*** are the corresponding eigenvectors of *K*. Spectral decomposition of the *K* matrix made it possible to estimate the variation captured by the first eigenvector, which helped determine the extent of the population structure among the breeding groups.

### Statistical models

#### Univariate polygenic model

The univariate, individual tree (“animal”) mixed model for each trait (*t*) was:
2

where *y*_*t*_ is a vector of phenotypic measurements at age 17, *b* is a vector of fixed block effects, *a*_*i*_ is the vector of additive genetic (breeding) values (EBVs), *e* is the vector of residual error effects, and *X* and *Z* are incidence matrices linking phenotypic records (*y*) to estimates of effects. Expectation of the model is *E*(*a*), *E*(*e*) = 0 and *E*(*y*) = *Xb*, with additive genetic and error effects distributed as  and  where *A* is the NRM describing the additive genetic relationships among individuals and *I* is an identity matrix.

#### Bivariate polygenic model

To test the assumption that a trait measured in different environments is affected by the genotype-by-environment interaction (GEI), the univariate model can be extended to its bivariate counterpart by stacking up the vectors for the two traits as:
3

where the total vectors of random effects have covariance structures var[(*α*_1_, *α*_2_)^T^] = *G* = *C* ⊗ *A* and var[(*e*_1_, *e*_2_)^T^] = *R* = *E* ⊗ *I* for the additive genetic and residual effects, respectively, where ⊗ is the direct (kronecker) product and *C* and *E* are matrices containing the additive genetic and residual (co)variance parameters to be estimated as:
4

which indicates that there is a genetic covariance across sites (with information flowing through *A*) but that the residual covariance across sites is zero because measures took place on different individuals in different environments.

Model parameters were sampled from the posterior distribution using the MCMCglmm v2.16 package [35] in R v2.15.2 [34]. Each model was run for 260,000 iterations with a burn-in of 60,000 and thinning interval of 200, resulting in 1,000 samples saved per chain. Fixed-effect priors were drawn from the default normal distribution with large (10^8^) variance. Weak, proper priors drawn from the inverse-Wishart distribution were set to the phenotypic variance divided by the number of random effects in the model with the degree of belief parameter set to 1 and 2 for uni- and bivariate models, respectively. Mixing and stationarity of the chains was assessed with trace plots of samples from the posterior distribution using the coda package [36]. Posterior distributions of the parameters were summarized using the mode and the highest probability density (HPD) interval (95%) with the MCMCglmm and coda packages. Posterior distributions of heritability (*h*^2^) and genetic correlations between traits (*r*_12_) were calculated as  and  respectively. Estimated breeding values ((*EBVs*; *â*_*i*_) obtained from the bivariate analyses were stored on the site where phenotypes were observed and were subsequently used to determine prediction accuracies with univariate SNP-based GEBVs (see next).

Following bivariate analyses, phenotypes were adjusted for fixed block effects and scaled by within-site phenotypic standard deviation in order to account for (small) differences in phenotypic variation for traits on different sites. No differences in genetic means between the breeding groups were detected. Phenotypes were then combined across sites for use in marker regressions, assuming that traits on different sites were a single trait with a genetic correlation equal to unity.

#### Marker regression models

In multiple-marker regression, many SNPs are simultaneously estimated as random effects in the individual tree model:
5

where *y*_*s*_ is a vector of adjusted and standardized phenotypic measurements at age 17, 1 is a vector of ones, *μ* is an intercept, *a*_*i*_ is the vector of additive genetic (breeding) values, *u*_*k*_ is a vector of random marker effects with the *n* x *m* incidence matrix containing marker covariates coded as *Z*_*ki*_ = (0, 1, 2) so that the sum of marker effects approximates the individual (additive) genomic estimated breeding value (GEBV)  and *e*_*i*_ is the vector of residual error effects. Additive genetic (polygenic) and error effects were assumed to be distributed as in eq. 2. The intercept was assigned a flat (uninformative) prior, whereas variance components were assigned a scaled inverse- *χ*^2^ prior density with degrees of freedom (df) and scale (S) parameters set to  for the error, additive genetic and marker effects, respectively, as recommended in [37].

Two types of marker regression were performed that differ in the prior distribution of SNP effects. In ridge regression (RR), all SNP effects are assumed to have a common variance by assigning a Gaussian prior as . Under this assumption all marker coefficients are shrunk to the same extent, which is appropriate for traits controlled by many genes of small effect, similar to the assumptions of the infinitesimal pedigree-based (polygenic) model. In contrast, the LASSO allows for marker-specific shrinkage effects, making it appropriate for traits thought to be under control of fewer genes of moderate to large effect. In the LASSO, the distribution of SNP effects are assigned a double exponential (DE) prior as  which states that the marker-specific prior is Gaussian, with marker-specific shrinkage of effects depending on *τ*^2^, which is in turn controlled by the prior distribution of the regularization parameter (*p*(*λ*)) [37]. The regularization parameter (λ), which allows the degree of shrinkage to be estimated from the data, was assigned a prior Beta distribution as outlined in [37], with parameters shape = 0.52 and rate = 1 × 10^−4^ yielding λ = 57.5 for our data. Large values of λ produce more informative (sharper) priors on marker effects [26], which will shrink (truly) small effects towards zero to a greater degree than in RR. Pedigree-based (A) best linear unbiased prediction (BLUP), ridge regression (RR) BLUP, and LASSO (L) regression were performed using the BLR package [38] in R v2.15.2 [34].

Single-marker regression (SMR; association testing) was also conducted to test the hypothesis that individual marker loci were in partial or complete linkage disequilibrium with a causal gene and to delineate subsets of markers for further marker regressions. Marker effects were treated as fixed effects (unlike in eq. 5) in SMR and relatedness structure was controlled using the numerator (*A*) or genomic relationship (*K*) matrix using the EMMAX algorithm [39] in the rrBLUP (v4.1) package [40] in R (v2.15.2) [34]. Variance components are estimated only once in a base model without SNPs using EMMAX and, subsequently, each SNP is added to the model in turn, equations are solved, and a *F*-test is constructed for each SNP to test the hypothesis that the SNP effect *u*_*k*_ ≠ 0.

Traits were modeled using 1) all individuals with all SNPs and/or the pedigree and 2) subpopulations of individuals with all or subsets of SNPs and/or the pedigree in cross-validation (CV). SNP subsets for CV were retained for reevaluation using RR in five different ways. First, the SNPs with the largest absolute effects (*P* < 0.05) using SMR were retained, controlling for relatedness with *A* or *K*. Next, the 600 SNPs with largest absolute effects were retained from the full model (all individuals and SNPs) using RR and pedigree information (FM-RRA), which approximated the maximum number of significant SNPs from SMR. SNP effects were estimated in a subpopulation (see below) and subsequently re-evaluated using RR in the same (e.g. BG1) or complementary subpopulation (e.g. BG1 → BG2). In the former case, SNP effects treated as fixed effects in SMR may be biased when estimated and validated in the same non-independent data [41, 42]. Finally, as controls, two sets of 600 SNPs were built by selecting SNPs randomly or by selecting those with the highest observed minor allele frequencies. For each of these controls, the selected SNPs remained the same across traits and subpopulations.

For cross-validation (CV) analyses, individuals (CV1) or full-sib families (CV2) were assigned to 10 folds randomly. In each of 10 rounds of CV, 9 folds acted as the training set in which SNP effects were estimated in order to predict the genetic values of individuals in the remaining fold, whose phenotypes had been withheld. For models tested across subpopulations (BG1 → BG2 or S1 → S2), that is training the models in one subpopulation and testing them in the other one, each CV round used 9 of 10 folds as the training set from subpopulation 1, with a different fold used as the testing set for each round of CV from subpopulation 2. Prediction accuracies were calculated as the correlation (cor (*ĝ*, *â*)) between the sum of CV SNP estimates (*ĝ*) or GEBVs and the additive genetic (breeding) value (*â*) or EBVs estimated using the bivariate, pedigree-based model and all phenotypic data, which was considered the best estimate of the “true” breeding value.

Subsets of individuals with mean training and testing set sizes of 787 and 87 individuals, respectively, were used to perform marker regressions and test prediction accuracy four different ways:

- BG1 (or BG2) – within-breeding group CV simulates a situation where related individuals have already been phenotyped on both (or similar) sites, as BG members were distributed across sites. GEI could lower prediction accuracy if present.
- BG1 → BG2 – between-breeding group CV removes all known relatedness between CV sets, establishes baseline accuracy due to (short-range) LD and thus tests if the model is transferable beyond the study material. As members of both BGs are present in both environments, GEI could have an impact.
- S1 (or S2) – within-site CV simulates a situation where models are trained by pooling individuals across BGs, with no known relatedness between BG. Evaluation occurs within site, thus GEI cannot affect the results.
- S1 → S2 – between-site CV simulates a situation where no phenotypes have been observed on related individuals in the second environment, testing the assumption that the traits on different sites have a genetic correlation of one.

## Results

Univariate heritabilities (not shown) were similar to their bivariate counterparts (Table 2), suggesting homogeneous phenotypic variance across sites. Although the genetic correlation (*r*_*12*_) was not equal to 1 across sites (Table 2), it was moderately high (for growth traits; *r*_*12*_ = 0.73), to high (for wood quality traits; *r*_12_ = 0.83). This implied that, biologically, the GEI was not strong enough to warrant the more complex (bivariate) model for wood traits (less so for growth traits) and that data might be pooled across sites and considered as a single trait.

**Table 2:** **Bivariate heritabilities (** ***h*** ^**2**^ **), genetic correlations (** ***r***_**12**_ **), and highest probability density interval (95%) for wood quality and growth traits considered as correlated traits when measured on different sites**

Site*		ADEN^†^	AMFA	HT17	DBH17
S1	*h* ^2^	0.33 (0.251, 0.585)	0.30 (0.169, 0.516)	0.39 (0.229, 0.640)	0.39 (0.172, 0.542)
S2	*h* ^2^	0.34 (0.181, 0.494)	0.32 (0.183, 0.510)	0.57 (0.378, 0.816)	0.32 (0.185, 0.561)
S1//S2	*r*_12_	0.83 (0.548, 0.919)	0.83 (0.570, 0.948)	0.73 (0.432, 0.879)	0.73(0.432, 0.879)

*S1: Asselin Township (S1-C); S2: St. Casimir (S2-D).

^†^ADEN: average wood density; AMFA: average microfibril angle; HT17: 17-year height; DBH17: 17-year diameter at breast height.

**Figure 1:**
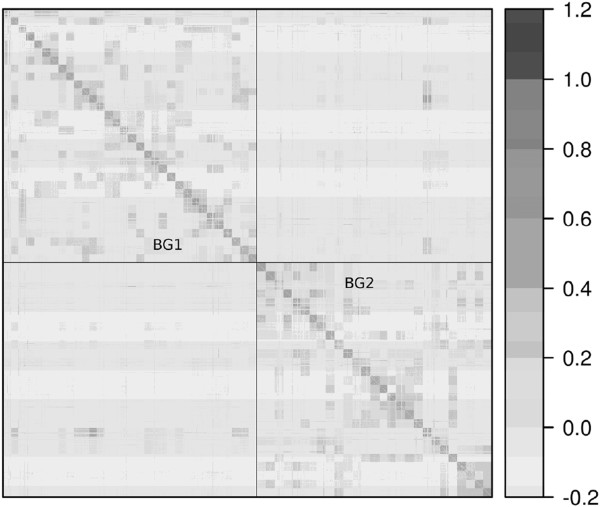
**The realized genomic relationship (** ***K*** **) matrix.** *K* matrix sorted by breeding group (BG) and full-sib family, with BG1 in the upper left and BG2 in the lower right.

Spectral decomposition of the *K* matrix revealed that < 5% of the variation was captured by the first eigenvector. This indicated that population structure was not important among the breeding groups and that *A* or *K* was sufficient to capture the relatedness among individuals. The lack of phenotypic differences observed between the two breeding groups, BG1 and BG2, supported this lack of structure. The *K* matrix captured a low level of relatedness between the two BGs (Figure 1), implying that some pedigree errors or cryptic relatedness between groups was present. The elements in *K* underestimated the corresponding non-zero, expected relationship values in *A*, with median (first and third quartiles: range) values for half- and full-sibs of 0.20 (0.15, 0.25; −0.12 – 0.90) and 0.44 (0.38, 0.49; −0.09 – 1.05), respectively.

### Full model with all SNPs

Modeling all individuals (*n* = 1,748) and SNPs (*m* = 6,932) simultaneously with ridge regression (FM-RR), which assumes a common variance across SNPs, only improved model fit (lower DIC) and reduced the error variance for ADEN, compared with the pedigree-based model (FM-A) (Table 3). For the other three traits (AMFA, HT17, DBH17), FM-RR resulted in poorer model fits and inflated error variance, signifying either that the pedigree contained valuable information not captured by the markers (despite some potential pedigree errors) and/or that markers were overfitting to spurious effects in the data. Comparing models with both pedigree and marker (FM-RRA) and pedigree-only (FM-A) information confirmed that the pedigree accounted for a large proportion of the variance, even with markers present in the model. However, both sources of information (FM-RRA) resulted in better fits and lower error variances compared with both the FM-A and the FM-RR models (Table 3), implying that markers were partially capturing non-redundant information to the pedigree.

**Table 3:** **Full-model posterior means of variance components using all SNPs (** ***m*** **= 6,932), all individuals (n = 1,748), and deviance information criterion (DIC) for wood quality and growth traits**

Trait^†^	Model*			***λ*** ^***2***^		DIC
ADEN	FM-A	0.33	–	–	0.67	4558
	FM-RR	–	1.7 × 10^−4^	–	0.65	4493
	FM-RRA	0.19	1.4 × 10^−4^	–	0.58	4433
	FM-L	–	–	88	0.65	4491
	FM-LA	0.18	–	90	0.57	4431
AMFA	FM-A	0.32	–	–	0.72	4660
	FM-RR	–	1.1 × 10^−4^	–	0.77	4679
	FM-RRA	0.21	7.5 × 10^−5^	–	0.69	4634
	FM-L	–	–	115	0.77	4275
	FM-LA	0.22	–	144	0.69	4633
HT17	FM-A	0.47	–	–	0.62	4608
	FM-RR	–	1.2 × 10^−4^	–	0.77	4740
	FM-RRA	0.37	5.4 × 10^−5^	–	0.61	4602
	FM-L	–	–	115	0.77	4739
	FM-LA	0.41	–	160	0.59	4577
DBH17	FM-A	0.29	–	–	0.76	4786
	FM-RR	–	9.1 × 10^−5^	–	0.83	4824
	FM-RRA	0.21	6.1 × 10^−5^	–	0.74	4769
	FM-L	–	–	134	0.83	4823
	FM-LA	0.23	–	158	0.72	4759
MSE^‡^		0.093	2.1 × 10^−5^	16.9	0.047	

^†^ADEN: average wood density; AMFA: average microfibril angle; HT17: 17-year height; DBH17: 17-year diameter at breast height.

*FM-A: Full model - pedigree-based; FM-RR: Full model - ridge regression with marker data; FM-RRA: Full model - combined ridge regression with markers and pedigree data; FM-L: full model - the least absolute shrinkage and selection operator; FM-LA: full model – combined least absolute shrinkage and selection operator, and pedigree information.

^‡^Mean standard error over models.

Allowing marker-specific shrinkage with the LASSO (FM-L and FM-LA) resulted in only very minor improvement in model fit and reduced error variance over the corresponding models using a common SNP variance (FM-RR and FM-RRA) for all traits (Table 3). The variance of the *λ* parameter increased from the starting values, indicating a tendency to shrink marker effects more severely towards zero (Table 3). Again, pedigree information proved important in the FM-LA model, allowing *λ* to further increase, providing even sharper shrinkage. Nevertheless, correlations between SNP effect estimates in complementary models (FM-L vs FM-RR, FM-LA vs FM-RRA) were very high (*r* ≥ 0.98, data not shown) and, given the limited improvement in LASSO fits, this model was not further considered.

### Cross-validation (CV1: individual assignment) with all SNPs

For CV1, prediction accuracies were high for pedigree-only (CV1-A) models because the CV sets were highly related. Marker-only models (CV1-RR) captured a high proportion (0.87 ≤ 0.92) of the CV1-A prediction accuracy for all traits, when modeled within breeding groups (BG1) or environment (S1) (Table 4). Modeling both pedigree and marker effects (CV1-RRA) led to prediction accuracies that approached but did not reach those obtained using the pedigree-only model, meaning that the previously seen better model fits for FM-RRA (Table 3) did not necessarily translate into better predictability in CV. This might have been due to the overfitting of marker effects and/or high multicollinearity between the marker and pedigree components. The same pattern was observed within BG2 and S2 (data not shown). Prediction accuracies were reduced somewhat for wood traits (ADEN, AMFA) across models when the training and testing sets were in different environments (S1 → S2) compared to evaluation within the same environment (S1, Table 4). However, this reduction in accuracy was much more pronounced for growth traits (HT17, DBH17), especially for DBH17. Prediction accuracies were near zero for all traits across models when training and testing sets were in different, genetically unrelated, breeding groups (BG1 → BG2, Table 4), as suspected from previous results with half-sib families [18].

**Table 4:** **Prediction accuracies for wood quality and growth traits estimated with cross-validation sets built with individuals within full-sib families using pedigree (CV1-A), ridge regression (CV1-RR) or both (CV1-RRA) sources of information with all SNPs (** ***m*** **= 6,932) and various subpopulations of individuals**

		Cross-validation			
Trait^†^	Subpopulation*	Pedigree (CV1-A)	Markers (CV1-RR)	Markers and pedigree (CV1-RRA)	Relative efficiency markers/pedigree (%)
ADEN	BG1	0.86	0.79	0.83	92
	BG1 → BG2	−0.02	0.06	0.22	–
	S1	0.83	0.75	0.80	90
	S1 → S2	0.72	0.66	0.71	92
AMFA	BG1	0.84	0.77	0.82	92
	BG1 → BG2	0.03	0.03	0.03	–
	S1	0.79	0.71	0.77	90
	S1 → S2	0.70	0.61	0.67	87
HT17	BG1	0.67	0.58	0.65	87
	BG1 → BG2	0.02	−0.10	−0.08	–
	S1	0.76	0.68	0.74	89
	S1 → S2	0.40	0.34	0.40	85
DBH17	BG1	0.60	0.52	0.58	87
	BG1 → BG2	−0.01	−0.16	−0.14	–
	S1	0.78	0.69	0.76	88
	S1 → S2	0.29	0.24	0.30	83
MSE^‡^		0.026	0.024	0.021	–

^†^ADEN: average wood density; AMFA: average microfibril angle; HT17: 17-year height; DBH17: 17-year diameter at breast height.

*BG1: both training and testing sets from breeding group 1; BG1 → BG2: training sets from breeding group 1 and testing sets from breeding group 2; S1: both training and testing sets from site 1; S1 → S2: training sets from site 1 and testing sets from site 2.

^‡^Mean standard error over cross-validation iterations and subpopulations.

### Cross-validation (CV2: family assignment) with all SNPs

Overall in cross-validation using family assignment (CV2) where relatedness between CV sets did not exceed those of half-sibs, prediction accuracies decreased sharply (e.g. 0.12 – 0.41 for BG1 and S1 with RR) for all models, traits and subpopulations (Table 5), compared with those in CV1, i.e. cross-validation where individuals were assigned to the various sets (Table 4). However, the same trend was also observed for the pedigree-based prediction (CV2-A) and as for CV1, the models based on markers only (CV2-RR) captured between 75% and 104% of the CV2-A prediction accuracy (Table 5). As CV1 and CV2 evaluate exactly the same set of individuals for BG1 and S1, differences in prediction accuracy can only be due to lower relatedness between CV sets, not to different LD patterns across different sets of individuals. That is, if markers were in LD with causal loci, prediction accuracies for marker-based models (CV2-RR) should be superior to those of pedigree-based models (CV2-A), which was not usually observed. Part of the lower prediction accuracy in CV2-RR was captured with CV2-RRA, i.e. when combining pedigree and marker information, meaning that even the weaker pedigree structure (i.e. half-sibs; some families across the training and testing sets sharing one of the parents but not both) provided useful information not captured when using markers only. Furthermore, this lower relatedness between training and testing sets in CV2 resulted in three times greater variability in prediction accuracy among CV folds (MSE, Table 5) than in CV1. Similar to CV1, CV2 prediction accuracies were higher within the same environment (S1) than when predicting between environments (S1 → S2), where prediction accuracies for growth traits were more reduced than those for wood traits (Table 5). As suspected, by removing all known relatedness between training and testing sets (BG1 → BG2), prediction accuracies for RR models fell to around zero for all traits. The fact that the expected prediction accuracy between BGs (BG1 → BG2) for the pedigree-only model (CV2-A) was close to zero indicates that sampling error might have contributed to small, non-zero prediction accuracies for models including markers, such as for wood density (Table 5).

**Table 5:** **Prediction accuracies for wood quality and growth traits estimated with cross-validation sets built with full-sib families, using pedigree (CV2-A), ridge regression (CV2-RR) or both (CV2-RRA) sources of information with all SNPs (** ***m*** **= 6,932) and various subpopulations of individuals**

		Cross-validation			
Trait^†^	Subpopulation*	Pedigree (CV2-A)	Markers (CV2-RR)	Markers and pedigree (CV2-RRA)	Relative efficiency markers/pedigree (%)
ADEN	BG1	0.57	0.59	0.62	104
	BG1 → BG2	0.10	0.10	0.19	–
	S1	0.71	0.63	0.69	89
	S1 → S2	0.59	0.53	0.57	90
AMFA	BG1	0.55	0.36	0.47	65
	BG1 → BG2	0.13	0.04	0.05	–
	S1	0.62	0.52	0.61	84
	S1 → S2	0.52	0.41	0.50	79
HT17	BG1	0.44	0.33	0.42	75
	BG1 → BG2	−0.01	−0.01	−0.01	–
	S1	0.66	0.55	0.64	83
	S1 → S2	0.33	0.22	0.29	67
DBH17	BG1	0.30	0.29	0.34	97
	BG1 → BG2	0.13	−0.06	−0.07	–
	S1	0.53	0.48	0.53	91
	S1 → S2	0.23	0.15	0.20	65
MSE^‡^		0.081	0.075	0.074	–

^†^ADEN: average wood density; AMFA: average microfibril angle; HT17: 17-year height; DBH17: 17-year diameter at breast height.

*BG1: both training and testing sets from breeding group 1; BG1 → BG2: training sets from breeding group 1 and testing sets from breeding group 2; S1: both training and testing sets from site 1; S1 → S2: training sets from site 1 and testing sets from site 2.

^‡^Mean standard error over cross-validation iterations and subpopulations.

### Cross-validation with SNP subsets

For SNP subsets that were identified and re-evaluated within the same population (BG1 or S1), all model-based methods used to select subsets performed relatively well (Table 6). SNP subsets selected for their largest absolute effects had accuracies slightly superior to those of randomly selected (CVSS_RAN_) or highest minor allele frequency (CVSS_HMAF_) subsets for growth traits, but differences for wood traits were less clear. Prediction accuracies were slightly higher when SNP effects had been treated as fixed effects (CVSS_A_ or CVSS_K_) and then re-evaluated in the same population (BG1 or S1). This trend suggests that, in some cases, completely eliminating many markers of supposedly zero effect did not hamper much the estimation of the fewer “true” marker effects, as was previously hypothesized from results obtained using the LASSO models. However, in many instances, the accuracy estimates were slightly lower than those obtained with the full set of markers. This might be due to the removal of some markers that were in LD with QTLs associated with the traits of interest.

**Table 6:** **Prediction accuracies for wood quality and growth traits estimated with cross-validation sets built with individuals within full-sib families for wood quality and growth traits using ridge regression (RR) for subsets (SS) of SNPs from single-marker regression controlling for** ***A*** **or** ***K*** **, the largest absolute effects from the full model (RRA), the highest minor allele frequencies (HMAF), and a random selection (RAN) for various subpopulations of individuals**

	Accuracies obtained in cross-validation with various marker subsets					
Trait^†^	Number of markers (***m***) and subpopulation*	Association tests using pedigree information(CVSS_A_)	Association tests using genotypic information^₸^(CVSS_K_)	Markers with largest absolute effects (CVSS_RRA_)	Markers chosen randomly (CVSS_RAN_)	Markers with highest minor allele frequencies (CVSS_HMAF_)
ADEN	*m*	640	404	600	600	600
	BG1	0.76	0.78	0.74	0.78	0.78
	BG1 → BG2	0.20	0.06	0.03	0.06	0.16
	S1	0.74	0.77	0.74	0.71	0.73
	S1 → S2	0.55	0.51	0.52	0.58	0.61
AMFA	*m*	406	359	600	600	600
	BG1	0.74	0.78	0.72	0.75	0.74
	BG1 → BG2	0.03	0.04	0.01	−0.12	0.11
	S1	0.73	0.76	0.69	0.65	0.66
	S1 → S2	0.51	0.54	0.44	0.55	0.52
HT17	*m*	476	426	600	600	600
	BG1	0.64	0.70	0.69	0.56	0.57
	BG1 → BG2	−0.03	−0.04	−0.07	0.00	−0.19
	S1	0.72	0.77	0.74	0.63	0.64
	S1 → S2	0.28	0.29	0.29	0.32	0.29
DBH17	*m*	482	403	600	600	600
	BG1	0.56	0.61	0.58	0.49	0.51
	BG1 → BG2	−0.12	−0.13	−0.25	−0.05	−0.23
	S1	0.71	0.77	0.70	0.63	0.65
	S1 → S2	0.17	0.18	0.18	0.22	0.20
MSE^‡^		0.025	0.024	0.023	0.024	0.023

Subset of significant SNPs (*P* < 0.05) identified after an association study using relatedness estimated with pedigree information.

^₸^Subset of significant SNPs (*P* < 0.05) identified after an association study using the realized genomic relationship matrix.

^†^ADEN: average wood density; AMFA: average microfibril angle; HT17: 17-year height; DBH17: 17-year diameter at breast height.

*BG1: both training and testing sets from breeding group 1; BG1 → BG2: training sets from breeding group 1 and testing sets from breeding group 2; S1: both training and testing sets from site 1; S1 → S2: training sets from site 1 and testing sets from site 2.

^‡^Mean standard error over cross-validation iterations and subpopulations.

When SNP subsets selected in BG1 were re-evaluated in a complementary subpopulation (BG2) with no known relatedness to BG1, prediction accuracies fell to near zero for all subsets and traits (BG1 → BG2, Table 6), similarly to what was observed with the full set of markers (Table 4). For models built on site S1 and evaluated in S2 (S1 → S2), prediction accuracies were moderate for wood traits (*r* > 0.44), but low for growth traits (*r* > 0.17, Table 6); again, they were lower than those obtained with all the markers (Table 4).

## Discussion

### Accuracy of GS models under various scenarios

It is clear from this and other studies on forest tree species e.g. [18, 19], that current moderately dense SNP panels can be used to capture additive genetic effects remarkably well under certain conditions. GS model accuracy is known to be affected strongly by *N*_*e*_ and marker density. For instance, it was shown for a white spruce training set of about the same size as the one used in the present study, and with similar marker density, that prediction accuracy of less than 0.40 could be achieved when the effective population size was over 600 [18]. However, although the number of SNPs in [18] and in this study was about the same, the number of distinct gene loci involved in the present study was more than twofold. This higher genome coverage might have also contributed slightly to improve accuracy estimates as expected from simulation studies for similar population sizes [4]. However, such improvement in accuracy appears to be modest at best, as indicated by accuracies obtained with subsets of random markers or with those with the highest MAF, which were almost the same as those estimated using all markers (Tables 4 and 6).

The prediction accuracy is also affected, to a lesser extent, by trait heritability and the training set size, once a minimum of *n* ≈ 1,000 – 2,000 individuals has been reached [4]. Given the number of SNPs (*m* = 6,932) and distinct loci (*l* = 6,918) used in this study and a white spruce genome size of ~ 2,100 cM [43], one would expect ~ 3.3 SNPs/cM if marker coverage was evenly distributed across the genome. At 2 – 3 SNPs/cM, *N*_*e*_ = 30 – 60 and a training set with *n* = 1,000 individuals, one should theoretically achieve GS model accuracies of 0.55 – 0.70 [4]. This was seen in this study (and surpassed in some cases) for predictions within breeding groups (N_e_ ≈ 20) or for the same environment when combining both breeding groups (N_e_ ≈ 40) using slightly smaller (mean *n* = 787) training sets. However, as for pedigree-based models, prediction accuracy of marker-based models decreased and fell near zero when close (CV2, Table 5) and all known relatedness (BG1 → BG2, Tables 4 and 5) was removed between the training and testing sets, respectively, similarly to what was observed in another GS study on a white spruce population of larger effective size [18]. This suggests that short-range LD, i.e. that between SNPs and causal loci still persisting in populations after several generations of random mating, might be more limited than expected, or that only a few of the SNPs used were in LD with causal loci (QTLs). Such an observation is reinforced by the trends in accuracy values seen when using subsets of random markers or those with the highest MAF, where accuracies did not decrease much compared with those estimated using all markers (Tables 4 and 6).

Low prediction accuracy between breeding groups in our study was not due to lack of modeling a group factor, as negligible phenotypic and genotypic differences were observed among breeding groups. This is consistent with the very weak genetic differentiation seen among white spruce populations in Québec [44, 45], making the 39 base parents in our study members of one large historical population. These base parents are still mostly unrelated, as spruce pedigrees in Québec have only been documented for two generations. In cattle, 50 K SNPs were deemed adequate to capture causal loci within breed, whereas 300 K SNPs were recommended for consistent cross-breed prediction accuracies, based on simulations and historical knowledge of *N*_*e*_ [46]. As expected, empirical cross-breed predictions in cattle fell to zero using 50 K SNPs, although combining breeds into a single training set resulted in prediction accuracies comparable to within-breed predictions [13]. At first glance, this appears analogous to predictions across and within breeding groups obtained in the present study, especially given that when we simultaneously considered both breeding groups when training the genomic prediction models, accuracies did not decrease much compared with those obtained by training models specific to each breeding group. When combining both breeding groups, a breeding group structure was introduced, although short-range LD should not differ between these breeding groups as they are believed to belong to the same ancestral population [18]. Any minor differences between breeding groups would have been absorbed by the marker effects thus biasing them, leading to prediction accuracies that may not be superior to modeling known subpopulation memberships [14].

### Reducing marker density and impact on prediction accuracies

Removing uninformative loci from the models appeared to have a positive, although not statistically significant effect on cross-validation for some traits within breeding group (BG1) or site (S1). Compared with the prediction accuracies obtained with all markers, a slight increase was indeed seen when using markers found significantly associated with traits when using either pedigree information (CVSS_A_) or realized genomic relationships (CVSS_K_), or markers with the largest absolute effects (CVSS_RRA_) (Table 6). This increase in accuracy can certainly be accounted for, at least in part, by the fact that these subsets of significant markers were not identified in an independent dataset [41, 42], and the accuracy estimates obtained could consequently be slightly biased upwards, thus accounting for the slight increase observed in accuracy. Such an increase in prediction accuracy was not observed for subsets of random markers, nor for those of highest minor allele frequency.

In a recent study on wood and growth traits in loblolly pine full-sib families, no increase in prediction accuracy was reported for various subsets of markers selected after association studies or randomly selected markers up to a maximum number of about 3,400 markers [20]. However, in another loblolly pine population that was clonally replicated, two different patterns were observed with regard to the influence of marker density on predictive ability [9]. For some traits such as growth, development and wood traits, the maximum value was reached with a few hundred markers and this value did not vary with a larger number of markers (total of 4,825 SNPs), whereas for traits such as wood density and disease-resistance-related traits, a small number of markers associated with these traits (100 to 500 markers) made it possible to reach the maximum value, which decreased with the addition of more markers. For wood density, the predictive ability obtained using subsets of several hundred markers was somewhat higher than, but not different from, subsets of randomly selected markers of similar size, as observed in the present study on white spruce. In a cattle population, genotypes from a 54 K SNP array made it possible to increase, on average, the prediction accuracies for daughter yield deviations by about 0.22 over those obtained with the pedigree information only, which represents an increase of about 60% [27]. Although removing uninformative SNPs did not reduce predictive ability estimates obtained with a 54 K SNP array, the high density arrays used allowed up to tens of thousands of SNPs to remain in the final model. These various results suggest that higher prediction accuracy might also be obtained in trees with subsets of markers significantly associated with the traits, but likely not until much higher density arrays than the current ones are available. The BayesCπ model [47] could be useful for single-step subset selection at higher marker densities because it appears to align more closely with the distribution of marker effects for wood and growth traits. BayesCπ simultaneously performs variable selection and shrinkage of SNP effects remaining in the model with a common variance, although empirical studies in plants have not proven it superior to the simpler RR model [9, 28].

### Capture of relatedness and long-range and short-range linkage disequilibrium

The observation that GS prediction accuracies sharply decreased when training and validation individuals were completely unrelated (BG1 → BG2) may be due to various causes. The first could be related to the fact that the long-range LD (co-segregating linkage blocks) generated through controlled crosses among the closely related individuals making up the training set may not be present in a non-related testing set. Linkage studies have shown that marker effects are generally limited to the family (genetic background) in which they were estimated [48] because the full variation of the causal loci in the population is not sampled. Linkage has nevertheless been found to be useful to increase genetic gain, even when no major effects were present, by increasing the precision of the relationship matrix [49]. In GS, where estimation occurs over the entire population, co-segregation of linkage blocks (long-range LD) was found to contribute significantly to prediction accuracy across full-sib families in maize [50]. In white spruce breeding groups such as those used in the present study, the contribution of long-range LD is also likely important, but it would decay more rapidly in subsequent generations than short-range LD [50]. However, combining full-sib families of several breeding groups could make it possible to develop GS models with high accuracies, as it was observed in the present study when individuals were pooled across breeding groups (scenarios S1 or S1 → S2). The persistency of that accuracy will depend on how quickly those linkage blocks decay over generations.

A complementary explanation for the loss of prediction accuracy among unrelated individuals could be that markers were largely capturing existing relationships between CV training and testing sets [15, 16]. It has been reported that genomic prediction accuracies can increase with the number of markers even in the absence of LD because markers capture additive relationships, but these accuracies will only be superior to the pedigree-based ones if markers are in LD with causal loci [12]. In line with [12], we observed in this study that the marker-based (RR) models implicating all markers or subsets of markers did not perform in most cases as well as the pedigree-based models using all the available trees or in most cases of cross-validation. Also in agreement with [12], prediction accuracies generally increased with the marker density used (*m* = 600 versus *m* = 6,932), but only slightly (Table 4: all markers versus Table 6: subsets of random markers). These observations imply that larger family sizes (*n* ≥ 100 individuals per family) might be needed in order to demonstrate the superiority of marker- over pedigree-based prediction accuracies [51] or that the contribution of LD to prediction accuracy was limited. For the former, more family members would allow SNP effects to be estimated more accurately, which would increase prediction accuracy [50]. The latter would mean that prediction accuracies are specific to a group of closely related individuals and may be overestimated because the larger contributions from relatedness and smaller contributions from short-range and long-range LD cannot be disentangled. This observation would argue in favor of GS models developed and implemented within the same population (or within a group of pooled populations, as discussed above) at least for the time being and considering the financial resources generally available to breeders.

One way to think about the level of LD in a population is to consider the relationship matrix estimated with the markers, which can be partitioned into 1) the expected (known) relationships obtained from pedigree information, 2) the deviations around these relationship values due to Mendelian sampling, i.e. those due to the fact that each offspring receives a different set of genes from its parents and that each offspring is different from its expected degree of kinship, and 3) the sampling error [52]. At high marker densities, the Mendelian sampling component can vary, for example, by as much as *a*_*ij*_ = 0.4 − 0.6 [53, 54] relative to the pedigree-based relationship coefficient (expected degree of kinship) of *a*_*ij*_ = 0.5 for full-sibs, and can be used to approximate the level of LD in a population [52]. Thus, to surpass pedigree-based prediction accuracies, it is clear that the precision of relatedness in the numerator relationship matrix (based on expected degree of kinship) needs to be increased. At high marker densities, genetic parameters can be estimated solely from Mendelian sampling components [53], implying that the genomic relationship matrix can be used to model the covariance among distantly related individuals [55], which establishes baseline accuracy due to LD [56]. In this case, however, large (training) population sizes would be required as the precision of quantitative genetic parameters is inversely proportional to the size of the estimated relatedness [55]. In our study, genomic relatedness likely underestimated pedigree-based relatedness, given that the scaling of the genomic relationship was appropriate. This observation suggests that many more markers would be necessary to obtain prediction accuracies superior to those using pedigree information. On the other hand, the “true” breeding values for wood and growth traits of the white spruce population considered in this study are not known and have been estimated using the pedigree information of the complete population and the bivariate individual-tree mixed model. One might think that “true” breeding values are closer to breeding values estimated with the realized genomic relationship matrix, which might influence the comparison of the pedigree-based and marker-based accuracies. As an attempt to answer this question, we re-estimated the accuracies replacing the “true” breeding values by the phenotypes, which are not influenced by the method of estimation as are the breeding values. For most of the scenarios tested, accuracies based on pedigree information were higher than either the genomic-based estimated breeding values or those estimated with combined genomic and pedigree information. Thus, this observation suggests that using the pedigree-based estimated breeding values as proxy for “true” breeding values has no significant impact on the estimation of prediction accuracies.

### Genotype-by-environment interaction

The results obtained with the two CV scenarios that tested the impact of GEI (BG1 and S1 → S2) were different for wood and growth traits. Wood traits were relatively unaffected by GEI in the bivariate model (*r*_12_ = 0.83), which was reflected in high (BG1) and moderately high (S1 → S2) prediction accuracies when phenotypes obtained in the second site (S2) were included or not in the training model, respectively (Table 4). These good prediction accuracies between environments would support the pooling of wood trait observations across sites and treat them as a single trait over environments. The situation might be different in more heterogeneous environments where GEI might be more important, as generally reported for western North America for instance [57], thus necessitating a careful case-by-case analysis.

Growth traits were more sensitive to GEI than wood traits, as prediction accuracies fell to moderate (BG1) and low (S1 → S2) levels, when phenotypes measured in the second site (S2) were included or not in the training model, respectively (Table 4). Such GEI was previously observed to be modest for growth traits in white spruce, though statistically significant [58]. This trend suggests that not only could a GEI component be beneficial in the statistical model, but information on relatives (markers or pedigree) alone is insufficient to predict genetic values in an environment where phenotypes have not been assessed.

Borrowing correlated information (phenotype of related individuals assessed on all experimental sites) to make predictions across environments was found useful in wheat [29], which may have been facilitated by the evaluation of homogeneous genetic (inbred) lines as opposed to the families or populations used in forestry, which are genetically heterogeneous. For both wood and growth traits, removing information quality (closeness of relatedness, Table 5) and quantity (number of markers, Table 6) between cross-validation sets weakened prediction accuracies, emphasizing the importance of close relatedness when making predictions across environments. When looking at growth traits in particular, it is encouraging to notice that the decrease in accuracy between sites was less pronounced for height than for DBH. Height is generally the preferred selection trait for fast growth by tree breeders [59]. This observation is in line with results reported for low GEI in white spruce for this trait [58], and also with the small reduction in accuracy observed across sites in a previous application of genomic selection to white spruce half-sib families in eastern Canada [18].

The bivariate model, which simultaneously considers the same trait measured on trees tested on two sites as different correlated traits, could be extended to a multivariate or reduced rank factor analytic model [29, 60] if there was an interest in fine-tuning the regional performance of seed orchards. Of course, this does not preclude combining traits over sites if overall performance in a large target environment is sought (selection of generalists instead of specialists). More importantly, it may be of interest to generate GEI for wood traits with silvicultural treatments [61] if interactions under intensive vs extensive management are anticipated.

### Implementation of genomic selection in spruce breeding programs

In practice, our results indicate that future prospects for genomic selection in spruces should take place within populations of small effective size combining several breeding groups, and thus implicating individuals of high relatedness within breeding groups. To recapitulate, this is first because marker-based model accuracies do not hold when removing relatedness between CV sets, indicating that with the current marker densities the overall short-range LD between markers and causal loci is either too low to be of practical use or not sufficiently well captured. With the proposed approach, reduction in marker coverage as shown in Table 6 would not have a significant negative impact on prediction accuracies, but as only long-range LD and relatedness are likely captured, prediction accuracy may not be persistent over many generations. Second, as expected, within-population prediction accuracies were high in this study, where effective population size was considerably constrained, and higher than those obtained in a much larger and genetically diverse spruce population with lower degree of relatedness [18]. However, it is clear that GS models could be developed for larger populations, as illustrated in the tested scenario S1, which combines breeding groups G1 and G2, without significantly affecting prediction accuracies (Table 4). Given the number of breeding groups tested, we could not determine what should be the most effective population size for a successful operational use of markers, but it is apparent that it could be at least 50, and maybe up to 100 [4], without losing too much prediction accuracy.

The obvious caveat for the proposed approach is that it is not possible to clearly separate the contributions of both relatedness and long-range LD (co-segregation) from prediction accuracy. Long-range LD may still be important if its contribution to prediction accuracy does not decay as fast as that due to relatedness, especially with recurrent selection, i.e. in a population with the same genetic background. The use of multigenerational data might be one way to disentangle the two sources of information.

A simple form of GS that does not depend on LD would consist of replacing the pedigree-based numerator relationship matrix with the genomic relationship matrix in the standard individual (animal) BLUP model [19], or to blend the two sources of information [62, 63], which is functionally equivalent to estimating and summing SNP effects using ridge regression [12, 51, 64]. We have shown that marker-based models could achieve, in most cases, almost 90% of the accuracy of pedigree-based models, and that data from breeding groups and sites could be combined without noticeably affecting the accuracy results. Based on these findings, we believe that kinship based on realized genomic relationships would be especially useful if pedigrees were unknown or incomplete (e.g. when using open or supplemental mass (pollimix) pollination in closed breeding orchards) to capture any cryptic relatedness not accounted for in the “known” pedigree (half-siblings only, for instance), to correct pedigree errors, and to overcome some of the strong assumptions of using the numerator relationship matrix in the pedigree-based BLUP [22]. This scenario would likely make it possible to obtain prediction accuracies that are higher than those obtained with incomplete pedigree information, and at the same time, to reduce breeding and testing costs. With the populations used in the present study, and ignoring knowledge on male contribution, the relative prediction accuracy of RR models over pedigree-based models (half-sib models) would have varied from 115% to 162%.

Another simple GS scenario that does not depend on our ability to separate the source of prediction accuracy would be to conduct genomic prediction within full-sib families [14]. In this case, the pedigree provides no information, LD would be high, and there would be no risk of linkage phases changing over families. The idea would be to generate large full-sib families (*n* = 200 and more), phenotype a fraction of the individuals (e.g. 25%), and select both phenotyped and unphenotyped individuals [14] for propagation. Although not tested in this study, the method could be useful in multiclonal forestry to identify superior genotyped seedlings derived from somatic embryos. For embryos originating from previously field-tested and genotyped members of the same cross, no additional field testing would be required. The difference between this method and genomic selection for recurrent selection is that the identified individuals would be propagated and, thus, marker effects tracing large linkage blocks would not need to persist in subsequent generations. Data could be pooled over families (and groups) as done in this study. With such a scenario and the strict assumptions under which this genetic gain potential might be realized, the breeding cycle for the set of families retained could be significantly reduced and gain per time unit more than double, i.e. the gain per year of breeding activities, which includes selection, crossing, and propagation phases (Table 7 and [18]).

**Table 7:** **Potential genetic gain as estimated empirically from a 5% selection intensity made within families using EBVs (based on pedigree information) and GEBV (based on all SNPs [** ***m*** **= 6,932]), and expected gain per time unit under the assumption that with use of markers and somatic embryogenesis, the breeding cycle could be reduced from 30 to 10 years by avoiding the 20-year field test and production phases**

		Empirical genetic gain				
Subpopulation*	Trait^†^	EBV_CV1_	GEBV_CV1_	EBV_CV1_/yr^a^	GEBV_CV1_/yr^b^	Ratio GEBV_CV1_/yr/EBV_CV1_/yr (%)
BG1	ADEN	27.79	24.31	0.93	2.43	262
	AMFA	−1.61	−1.31	0.05	0.13	245
	HT17	44.51	37.26	1.48	3.73	251
	DBH17	4.15	3.29	0.14	0.33	237
S1	ADEN	25.23	21.82	0.84	2.18	259
	AMFA	−1.72	−1.51	0.06	0.15	263
	HT17	43.43	38.36	1.45	3.84	265
	DBH17	9.27	8.70	0.31	0.87	282

*BG1: both training and testing sets from breeding group 1; S1: both training and testing sets from site 1.

^†^ADEN: average wood density; AMFA: average microfibril angle; HT17: 17-year height; DBH17: 17-year diameter at breast height.

^a^EBV_CV1_/T_C_, where T_C_ =30, and T_C_ is the number of years to complete a breeding cycle with conventional breeding methods.

^b^GEBV_CV1_/T_E_, where T_E_ =10, and T_E_ is the number of years to complete a breeding cycle with genomic selection and somatic embryogenesis to propagate the superior selected material.

## Conclusion

In the short term, genomic selection would be most useful to select superior individuals within large full-sib families. These individuals would be reproduced by vegetative propagation techniques such as somatic embryogenesis (SE), or a mix of SE and rooted cuttings, in order to develop a multivarietal forestry program. With regard to genomic selection for recurrent selection at current marker densities in spruce populations, its efficiency would depend on the presence of close relatives in order to exploit the capture of relatedness and supposedly long-range LD. Thus, a simple marker-based approach that aim to uncover relatedness when pedigrees have not been recorded or are only partially known would seem to be the most cost-effective application of this technology. For such a scenario, the total number of SNPs required would be only a few hundreds to a few thousands. In the longer term, superior marker-over pedigree-based predictions may be achievable in descendant generations of closed populations as short-range LD increases and pedigrees provide less information. However, to achieve this goal, denser marker arrays, more individuals per family, and more sophisticated experiment designs and genomic selection models [17] than those used in the current study will likely need to be considered. A step towards achieving this goal in forest genetics might also be the estimation across generations in order to better evaluate the persistency of marker effects.
